# An Observational Study With the Janssen Autism Knowledge Engine (JAKE^®^) in Individuals With Autism Spectrum Disorder

**DOI:** 10.3389/fnins.2019.00111

**Published:** 2019-02-27

**Authors:** Seth L. Ness, Abigail Bangerter, Nikolay V. Manyakov, David Lewin, Matthew Boice, Andrew Skalkin, Shyla Jagannatha, Meenakshi Chatterjee, Geraldine Dawson, Matthew S. Goodwin, Robert Hendren, Bennett Leventhal, Frederick Shic, Jean A. Frazier, Yvette Janvier, Bryan H. King, Judith S. Miller, Christopher J. Smith, Russell H. Tobe, Gahan Pandina

**Affiliations:** ^1^Neuroscience Therapeutic Area, Janssen Research & Development, Titusville, FL, United States; ^2^Computational Biology, Discovery Sciences, Janssen Research & Development, Beerse, Belgium; ^3^Statistically Speaking Consulting, LLC, Chicago, IL, United States; ^4^Informatics, Janssen Research & Development, Spring House, PA, United States; ^5^Statistical Decision Sciences, Janssen Research & Development, Titusville, NJ, United States; ^6^Computational Biology, Discovery Sciences, Janssen Research & Development, Spring House, PA, United States; ^7^Departments of Psychiatry and Behavioral Sciences, Duke Center for Autism and Brain Development, Duke University School of Medicine, Durham, NC, United States; ^8^Department of Health Sciences, Northeastern University, Boston, MA, United States; ^9^Department of Psychiatry, School of Medicine, University of California, San Francisco, San Francisco, CA, United States; ^10^Center for Child Health, Behavior and Development, Seattle Children's Research Institute, Seattle, WA, United States; ^11^Department of Pediatrics, University of Washington, Seattle, WA, United States; ^12^Eunice Kennedy Shriver Center and Department of Psychiatry, University of Massachusetts Medical School, Worcester, MA, United States; ^13^Department of Developmental-Behavioral Pediatrics, Children's Specialized Hospital, Toms River, NJ, United States; ^14^Center for Autism Research, Perelman School of Medicine, Children's Hospital of Philadelphia, University of Pennsylvania, Philadelphia, PA, United States; ^15^Southwest Autism Research & Resource Center, Phoenix, AZ, United States; ^16^Department of Outpatient Research, Nathan Kline Institute for Psychiatric Research, Orangeburg, NY, United States; ^17^Neuroscience Therapeutic Area, Janssen Research & Development, Pennington, NJ, United States

**Keywords:** autism spectrum disorder (ASD), biosensor, web and mobile application, mood report, assessment

## Abstract

**Objective:** The Janssen Autism Knowledge Engine (JAKE®) is a clinical research outcomes assessment system developed to more sensitively measure treatment outcomes and identify subpopulations in autism spectrum disorder (ASD). Here we describe JAKE and present results from its digital phenotyping (My JAKE) and biosensor (JAKE Sense) components.

**Methods:** An observational, non-interventional, prospective study of JAKE in children and adults with ASD was conducted at nine sites in the United States. Feedback on JAKE usability was obtained from caregivers. JAKE Sense included electroencephalography, eye tracking, electrocardiography, electrodermal activity, facial affect analysis, and actigraphy. Caregivers of individuals with ASD reported behaviors using My JAKE. Results from My JAKE and JAKE Sense were compared to traditional ASD symptom measures.

**Results:** Individuals with ASD (*N* = 144) and a cohort of typically developing (TD) individuals (*N* = 41) participated in JAKE Sense. Most caregivers reported that overall use and utility of My JAKE was “easy” (69%, 74/108) or “very easy” (74%, 80/108). My JAKE could detect differences in ASD symptoms as measured by traditional methods. The majority of biosensors included in JAKE Sense captured sizable amounts of quality data (i.e., 93–100% of eye tracker, facial affect analysis, and electrocardiogram data was of good quality), demonstrated differences between TD and ASD individuals, and correlated with ASD symptom scales. No significant safety events were reported.

**Conclusions:** My JAKE was viewed as easy or very easy to use by caregivers participating in research outside of a clinical study. My JAKE sensitively measured a broad range of ASD symptoms. JAKE Sense biosensors were well-tolerated. JAKE functioned well when used at clinical sites previously inexperienced with some of the technologies. Lessons from the study will optimize JAKE for use in clinical trials to assess ASD interventions. Additionally, because biosensors were able to detect features differentiating TD and ASD individuals, and also were correlated with standardized symptom scales, these measures could be explored as potential biomarkers for ASD and as endpoints in future clinical studies.

**Clinical Trial Registration:** https://clinicaltrials.gov/ct2/show/NCT02668991 identifier: NCT02668991

## Introduction

Widely accepted, standardized tools for diagnosing autism spectrum disorder (ASD) include the Autism Diagnostic Observation Schedule, 2nd edition (ADOS-2) and the Autism Diagnostic Interview-Revised (Falkmer et al., 2013). However, physiological and psychological instruments designed to detect change over time in core and associated symptoms of ASD are lacking. As ASD is a neurodevelopmental disorder with its roots in brain structure and function, it is reasonable that physiological and psychological measurements that assess this structure and function would be useful. In addition, monitoring of ASD symptoms in naturalistic settings will likely both improve care of people with ASD and yield insights into the condition. Such tools are needed to address significant unmet medical needs for improved diagnosis and expanded treatment options in ASD, and to develop novel therapies that target core and associated symptoms.

Global efforts to identify potential biomarkers for use in ASD research have been noted in the European Union (EU) and US. The EU-AIMS initiative consists of a large public-private partnership between academia, pharma, and foundations (Loth et al., 2016) to carry out several large-scale studies involving children, adolescents, and adults. In the US, the Autism Biomarkers Consortium Clinical Trial (ABC-CT) is engaged in a similar effort to examine change over time in a younger cohort of children and adolescents with ASD (McPartland, 2018). These large-scale consortia utilize a select group of expert academic sites and employ high-density tools (e.g., 128 lead electroencephalogram [EEG] and magnetic resonance imaging) to help identify biomarkers to stratify subgroups of individuals with ASD that might be more responsive to a particular therapy, and to measure change in symptoms and behavior over time.

### Biomarker Development in ASD

Biosensor research is relatively new in its development and application to ASD. Sensor-based biomarkers (e.g., EEG and eye tracking) have the potential to measure progress objectively and facilitate development of novel therapies for ASD core and associated symptoms (Murias et al., 2018). Biomarkers may be used to characterize participants who share common pathophysiology, as well as being an objective measure for clinical outcomes (Loth et al., 2017).

### Wearable Biosensors

#### Actigraphy

An actigraph measures physical activity and can be worn on a participant's wrist like a watch. Data recorded during the day can be used to identify time periods of sedentary, light, moderate, and moderate-to-vigorous activity. An ASD population is expected to spend more time engaged in sedentary activity, less time exposed to ambient light, and less moderate and moderate-to-vigorous activity than typically developing (TD) children of the same age (Vaughan Van Hecke et al., 2009). Additionally, it has been reported that sleep problems occur in ~50–80% of children with ASD, compared with 9–50% for a TD group (Richdale and Schreck, 2009). The use of actigraphy for sleep monitoring, with ~90% sensitivity of sleep estimation using curated data approaches, provides a valid, more tolerable, and efficient potential alternative to polysomnography (PSG) (Meltzer et al., 2012). Although PSG, of course, remains the gold-standard for sleep measurements, we feel the far greater versatility and practicality of actigraphy makes it a reasonable choice for use in clinical studies. The ability to automatically detect repetitive motor movements using actigraphy and machine learning has also been demonstrated (Goodwin et al., 2011, 2014; Großekathöfer et al., 2017).

#### Electrodermal Activity

ASD is partially characterized by sensory differences and atypical affective responses, symptoms included in standard diagnostic tools such as the ADOS-2 (Lord et al., 2012), Autism Diagnostic Interview (Lord et al., 1994), and the Diagnostic and Statistical Manual of Mental Disorders, 5th Edition (DSM-5) (American Psychiatric Association, 2013). Dysregulated emotional and atypical physiological responses may underlie these features (Woodard et al., 2012; Mazefsky et al., 2013; Levine et al., 2014; Klusek et al., 2015; Lydon et al., 2016). Similarly, individuals with ASD often demonstrate difficulties regulating their internal emotional states (Mazefsky et al., 2013). As such, electrodermal activity (EDA), a peripheral index of sympathetic nervous system arousal (Boucsein, 2012), may be informative in ASD populations (McCormick et al., 2014; Prince et al., 2017). For instance, processing of social stimuli may differentiate ASD and TD populations in terms of autonomic nervous system responsivity (Louwerse et al., 2014).

### Lab-Based Biosensors

Multiple brain systems are implicated in the formation and maintenance of core deficits in ASD. Establishing peripheral, non-intrusive methods such as EEG or eye tracking to interrogate these deficits would allow for *in vivo* study and potentially quantify ASD core deficits, as well as links to underlying ASD neurobiology (Black et al., 2017). EEG may be well-suited for detecting the alterations in brain connectivity found in ASD, through the use of coherence measurements and other paradigms (O'Reilly et al., 2017). Translational research suggests direct links between ASD neurobiology and social deficits. For example, EEG has been utilized in mouse models of Fragile X to link to neurobiological substrates (Goswami et al., 2019). Significant differences in EEG activity have been widely demonstrated in studies to distinguish individuals with ASD from TD controls (Gurau et al., 2017). Studies using eye tracking and event-related potentials (ERPs) to examine emotional face processing suggest that the neural correlates of gaze direction processing may be delayed in young children with ASD (Grice et al., 2005; Wang et al., 2013; Shou et al., 2017), and that individuals with ASD show an atypical pattern of emotional face processing and a reduced relationship between gaze behavior and neural processing of faces (Wagner et al., 2013).

Visual information processing in TD individuals is reinforced by additional speech information, demonstrating that TD children's ability to integrate multimodal input enables faster encoding and recognition of faces (Bahrick, 1987; Carpenter et al., 1998; Lydon et al., 2016). There is also evidence that face detection is facilitated when combined with directed speech in TD infants (Yirmiya et al., 1989). In contrast, children with ASD are characterized by limited attention to faces combined with under-responsivity to speech (Dawson et al., 2004; Wetherby et al., 2004; Chawarska et al., 2012). Limited attention to faces in individuals with ASD is particularly prominent when viewing dynamic videos presented in complex naturalistic contexts (Speer et al., 2007). It is possible that such limited attention to faces is a direct result of increased salience of objects, which are of high autism interest (HAI) (Senju et al., 2004; Sasson et al., 2008, 2011). It is also expected that differences between ASD and TD populations become apparent when dyadic cues consisting of directed speech and eye contact are introduced (Chawarska et al., 2016; Wang et al., 2018). Individuals with ASD assessed by eye tracking show diminished attention to scenes where an actress emulates bids for dyadic engagement, and spend less time monitoring the speaker's face in general and mouth in particular. Instead, their attention is directed toward toys as well as hand/action areas (Chawarska et al., 2012).

In contrast, when passively observing the interactions of others, children with ASD exhibit diminished attention to others' functional play activities, in addition to limited attention to faces (Shic et al., 2011). In combination, these studies provide evidence that eye tracking can identify and measure variables associated with limited social information processing in ASD, limitations likely reflecting fundamental social deficits in ASD and difficulties in subsequent social learning (Bushwick, 2001; Klin et al., 2003). Several studies have also suggested that eye tracking can be used to monitor the effects of pharmacological agents. For example, eye tracking studies have shown that individuals with ASD look more at eyes subsequent to administration of intranasal oxytocin (Andari et al., 2010; Auyeung et al., 2015); another study found increased orienting to biological motion subsequent to administration of a novel V1a antagonist (Umbricht et al., 2017). Murias et al. used eye tracking to demonstrate that improvements in social behavior were correlated with increased attention to social stimuli (Murias et al., 2018).

In the TD population, facial expressions activated via automatic or intentional mimicry appear to influence corresponding emotions, whereas the influence is impaired in the ASD population (Tomasello et al., 2005). Consequently, it is expected that individuals with ASD will show fewer facial emotional reactions (i.e., less variance in emotion) compared to TD individuals in response to videos designed to elicit overt emotional states, such as humorous or funny videos. A more neutral flatness of affect has been found in the activated facial expressions of children with autism (Yirmiya et al., 1989). It is also expected that facial expressions activated in the ASD population will be diminished compared to those of TD participants.

Finally, as suggested above, recent large scale trials (Bazelmans et al., 2018), and systematic reviews (Klusek et al., 2015; Lydon et al., 2016) of the literature on physiological reactivity to sensory, social, and emotional stimuli indicate that differences in cardiovascular arousal exist for many individuals with ASD.

### Deep Phenotyping Tools in ASD

ASD interventions are frequently evaluated using caregiver-reported measures administered during study visits. Two common caregiver measurements of behavior change in ASD are the Aberrant Behavior Checklist–Community (ABC) (Aman et al., 2004; Aman and Singh, 2017) that measures general behaviors, and the Social Responsiveness Scale 2™ (SRS-2) that measures symptoms associated with social behaviors (Constantino et al., 2003). While informative, these surveys are retrospective, which can reduce rating accuracy. For example, caregivers may report ASD symptoms as being worse in the past compared to real-time reporting, even in the absence of an intervention (Jones et al., 2015). Deficits in social communication (SC) may be a risk factor for the development of mood and anxiety disorders in ASD (De-la-Iglesia and Olivar, 2015; Gotham et al., 2015). Some interventions directed at SC (6 of 10 tested), led to improvements in depression and anxiety symptoms in ASD (Rumney and MacMahon, 2017). Understanding relationships between mental health and core ASD symptoms could help identify specific interventions to improve quality of life for individuals with ASD and their caregivers.

The Janssen Autism Knowledge Engine (JAKE®) system is an initiative to standardize physiological and psychological instruments to reliably identify and measure core and associated symptoms of ASD. A preliminary study was performed with 29 participants with ASD to test the feasibility of the JAKE system and the learnings regarding logistics, data collection, data quality, and analysis have been published (Ness et al., 2017). The results obtained for the performance of the system allowed for further refinement and validation of JAKE in this follow-up study in a completely independent sample. As compared to the initial study the system was complete and able to gather useable data. System reliability, quality, user feedback, and representative examples of results from the various components of JAKE are described herein as well as participant demographics and safety. Detailed methodology and results of the ABI, other components of My JAKE, and the various sensors and tasks are published elsewhere (Bangerter et al., 2017, In press; Manfredonia et al., 2018; Manyakov et al., 2018; Jagannatha et al., In press) or are in preparation.

## Materials and Methods

### Study Design

JAKE is a dynamically updated clinical research system developed to provide quantifiable and reproducible measures for use in assessing treatment outcomes, potentially including detection of change in ASD symptoms and ASD subgroup identification. JAKE is a three-part investigational system consisting of: My JAKE (a web and mobile application for use by caregivers and clinicians to log symptoms, record treatments, track progress, and gather comprehensive medical information); JAKE Sense (research biosensors and tasks designed to detect and monitor changes in experimental, proof-of-concept ASD biomarkers); and JAKE Stream (a system designed to collect, time-synchronize, and process data from both My JAKE [My JAKE Data Pipeline] and JAKE Sense [JAKE Sense Data Pipeline]).

**Figure 1:**
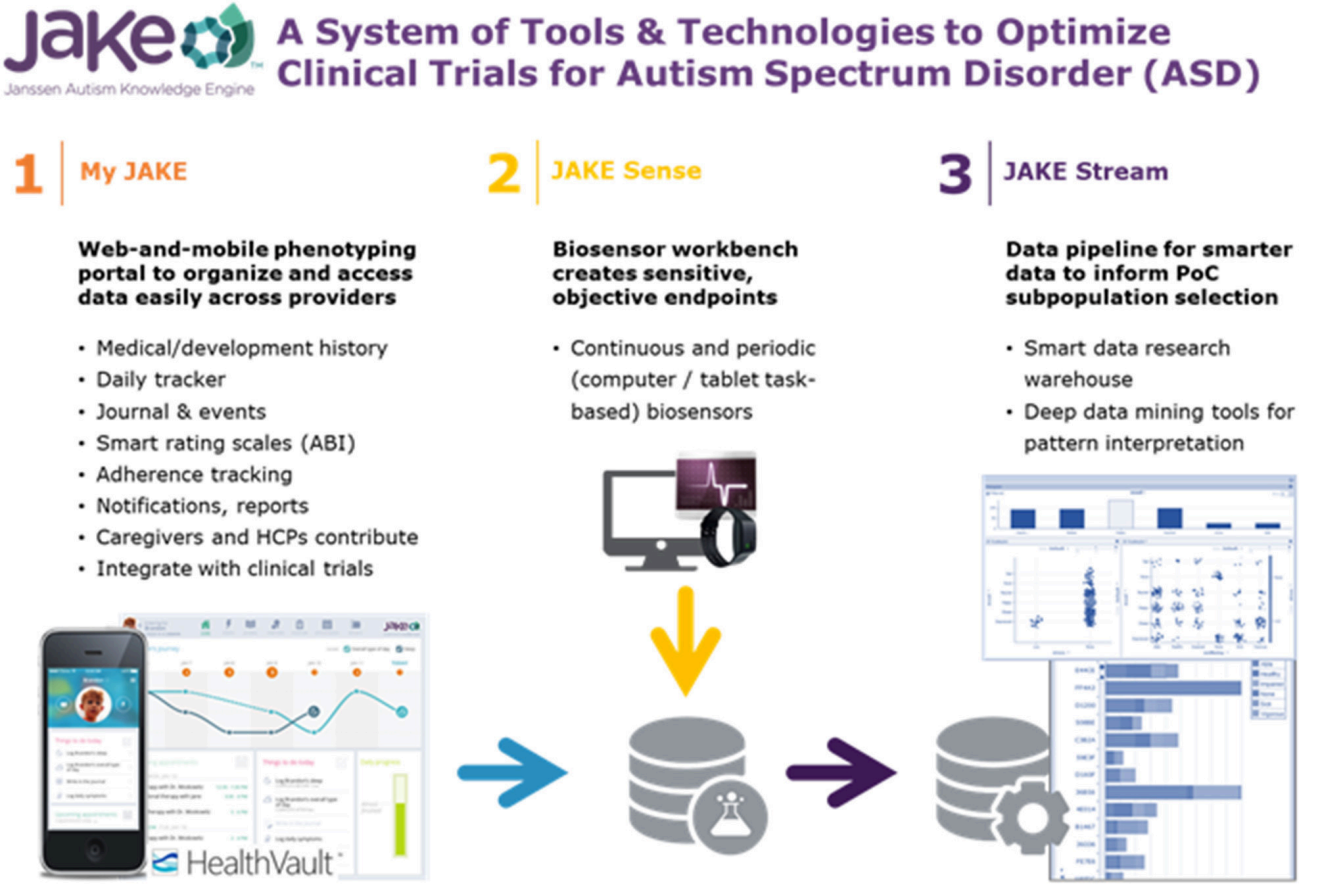
The JAKE system. ABI, Autism behavior inventory; HCP, health care professional.

This prospective, observational, non-interventional study was conducted from 06 July 2015 to 14 October 2016 at 9 study sites in the US and was completely independent from a previous study on the use of JAKE (Ness et al., 2017). The study consisted of a 14-day screening phase followed by an 8-to-10-week data-collection phase. Study visits were performed at baseline, Week 4, and study endpoint (8–10 weeks). Evaluations throughout the study encompassed several categories within the JAKE system (Figure 1). Though this was a non-interventional study, participants received treatment as usual, and there was some anticipated change in reported behaviors measured at baseline and endpoint. Observed improvements or worsening of behavior over the course of the study were used to assess JAKE's ability to detect change. This study was registered with clinicaltrials.gov (NCT02668991).

### Participants

The study enrolled males and females aged ≥6 years with an ADOS-2-confirmed diagnosis of ASD. Participants were permitted to receive behavioral and/or pharmacologic treatments for ASD and comorbid disorders during the study, but this was not required. Participants lived with a parent or primary caregiver (or a guardian or support provider, referred to as “caregiver” throughout this publication) or, if not, spent at least 3 h a day for at least 4 days each week or at least 3 weekends a month with a parent or primary caregiver. Key exclusion criteria were a measured composite score on the Kaufmann Brief Intelligence Test-2 (KBIT-2) (Kaufman, 2004) of < 60 during screening (or other recent IQ evaluation), history of or current significant medical illness, and documented psychological and/or emotional problems.

The study also enrolled a TD cohort comprised of males and females, aged ≥6 years, with a score in the normal range on the Social Communication Questionnaire (SCQ) (Rutter et al., 2003), no mental disorders as defined in the DSM-5, no significant medical illness, or current psychotropic medication. The TD cohort provided normative data for comparison with ASD participants, completed standard instruments and scales, a paper version of the ABI, and lab-based JAKE Sense assessments, but did not use My JAKE. Planned study cohorts are presented in Table 1.

**Table 1:** Planned study cohorts.

**ASD Cohort**	**TD Cohort**
Approximately 150 individuals with ASD aged ≥6 years No requirements or restrictions regarding concurrent therapies or treatments	Approximately 30 normally developing individuals with ASD aged ≥6 years, with ~5 individuals in each subgroup of ages 6–9 years, 10–12 years, 13–17 years, and ≥18 years
A 14-day screening phase, and an 8–10-week data collection phase extending from Day 0 (Baseline) to endpoint was planned	Single visit and single session with JAKE Task Battery and JAKE Sense
Optional: After the endpoint visit, participants may continue to use My JAKE and the Microsoft® HealthVault pHR to contribute data to the study until the end of the study	

*ASD, autism spectrum disorder; JAKE, Janssen Autism Knowledge Engine; pHR, personal healthcare record*.

Institutional Review Boards approved the study protocol and its amendments. The study was conducted in accordance with the ethical principles originating in the Declaration of Helsinki, consistent with Good Clinical Practices and applicable regulatory requirements. Participants, their caregivers (for participants < 18 years old), or legally authorized representatives provided written informed consent before participating in the study. The study is registered at clinicaltrials.gov (NCT02668991).

### Study Assessment Instruments and Scales

Throughout the study, data collected through My JAKE and JAKE Sense were compared against psychometrically standardized scales (completed outside the JAKE system) to assess validity of the system as a tool for measuring clinical outcomes in ASD (Table 2).

**Table 2:** Study assessment instruments and scales.

**Test**	**Description**
**DIAGNOSTIC AND CLASSIFICATION INSTRUMENTS ADMINISTERED AT SCREENING**^**a**^	
Autism Diagnostic Observation Schedule, 2nd edition (ADOS-2)	Used to accurately assess and diagnose autism spectrum disorders across age, developmental level, and language skills in ASD participants
Kaufman Brief Intelligence Test, Second Edition (KBIT-2)	A validated test used to obtain a quick estimate of intelligence, administered at screening in order both to help select participants capable of performing the required tasks and for analysis of data in ASD participants
The Mini International Neuropsychiatric Interview 7.0 (MINI 7.0) or pediatric component (MINI KID)	Used to rule out any major psychiatric diagnosis in TD participants, and to identify any psychiatric comorbidities in ASD participants
Social Communication Questionnaire, current form (SCQ)	A 40-item scale that evaluates social functioning and communication skills over the last 3 months, administered at screening. It was administered only to the TD cohort, to help rule out ASD
**STANDARD INSTRUMENTS FOR MEASURING CHANGE**^**b**^	
Aberrant Behavior Checklist (ABC)	A 58-item behavior rating scale, used to measure behavior problems across 5 subscales: irritability, lethargy (social withdrawal), stereotypy, hyperactivity, and inappropriate speech (Aman et al., 2004; Aman and Singh, 2017)
Zarit Burden Interview–Short Version (ZBI)	A 22-item scale assessed the psychological burden experienced by a caregiver, for both patients with dementia and children and adults with ASD (Zarit et al., 1980; Cadman et al., 2012)
Social Responsiveness Scale 2 (SRS-2)	Distinguishes autism spectrum conditions from other child psychiatric conditions by identifying the presence and extent of autistic social impairment (Constantino et al., 2003)
Child Adolescent Symptom Inventory – Anxiety (CASI-Anx)	A 21-point anxiety scale used as a possible outcome measure for autism (Sukhodolsky et al., 2008; Hallett et al., 2013)
Repetitive Behavior Scale—Revised (RBS-R) (caregiver)	A 43-item report scale used to indicate occurrence of repetitive behaviors and degree to which a behavior is a problem (Bodfish et al., 1999).

a Instruments used to establish the diagnosis of autism or help rule it out, to help classify participants by intelligence quotient (IQ), or to rule out or rule in the presence of other psychiatric disorders in participants.

b *Caregiver-reported rating scales used to assess change over time in particular domains of ASD symptoms and to validate components of the JAKE system. They were administered only to ASD participants at baseline, midpoint and endpoint visits. Scales were selected based on previous use in clinical trials for ASD, or recommendations in reviews of scales for use in measuring change in ASD core and associated behavior (Lecavalier et al., 2014; Anagnostou et al., 2015; Scahill et al., 2015) ASD, autism spectrum disorder; TD, typically developing*.

### Deep Phenotyping Tools-My JAKE

My JAKE is a web and mobile application (iOS+/Android+) consisting of various modules to help caregivers and healthcare providers who support individuals with ASD to log symptoms, demarcate events of interest, record treatments and medical information, and track overall study progress. My JAKE was developed, in part, to enhance the ability to understand the phenotypic underpinnings of individuals with ASD and to address both a lack of appropriate measures and convenient methods of use. The purpose of My JAKE is to provide a robust and sensitive set of integrated outcome measures for ASD clinical trials and other interventional studies in place of or in addition to current measures that do not necessarily assess the full range of ASD symptoms or were not designed to measure change over time.

All data created and accessed by My JAKE were saved to a caregiver's Microsoft HealthVault account, a publicly-available Class 1 electronic personal health record system. As the sole storage mechanism for all My JAKE data, the use of HealthVault permitted caregivers to own and control their dependent's study data, even after the study ended. Additionally, caregivers controlled which people and applications could access their HealthVault account at any time. The My JAKE application server is hosted on Microsoft Azure.

**Figure 2:**
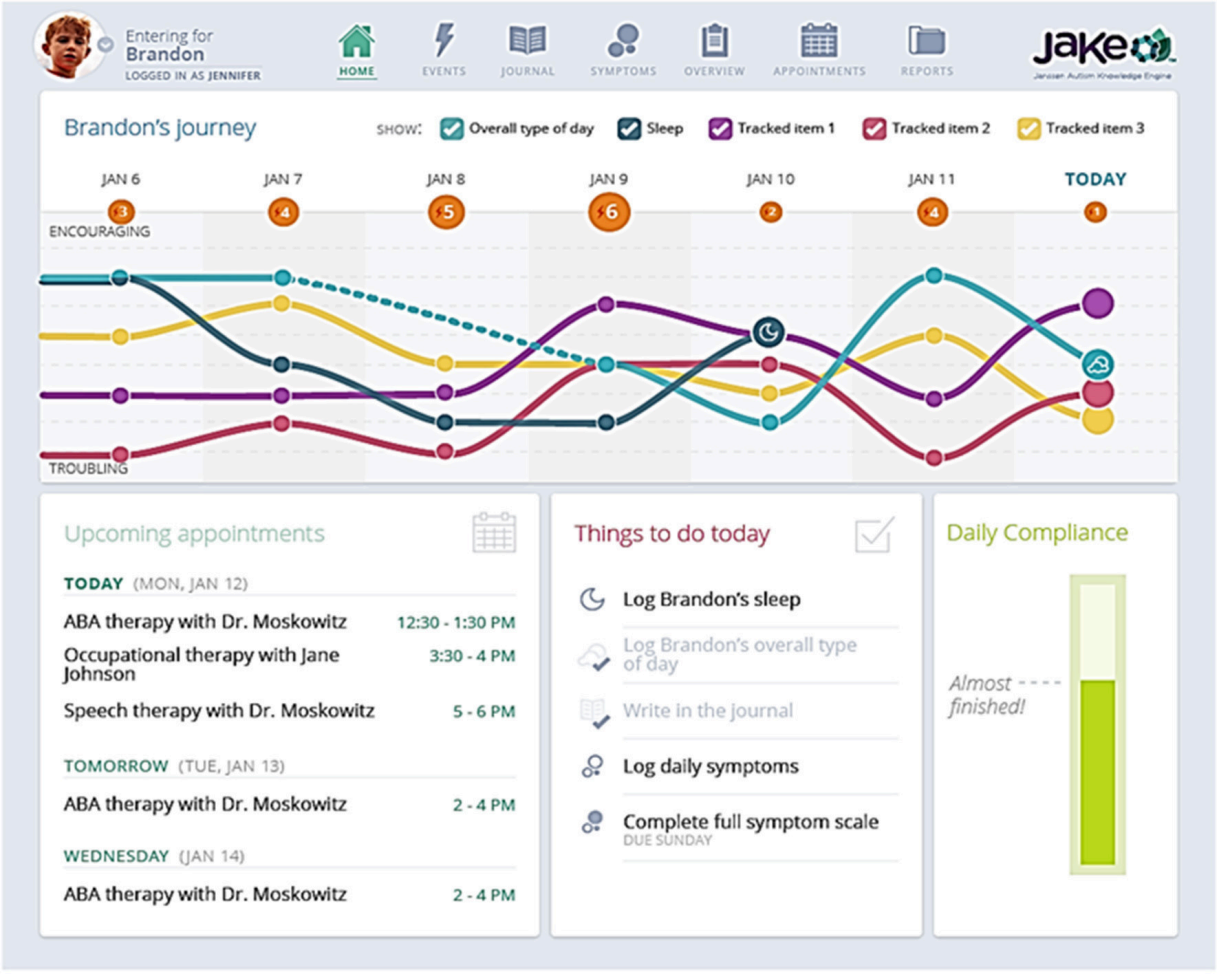
Sample My JAKE home page. ABA, Applied behavior analysis.

My JAKE is accessible through most web browsers, as well as mobile devices, and was used throughout the present study. The My JAKE home page includes a “Journey” chart for visualizing change of ASD “events” and selected symptoms, a study tasks tracker (the “To-Do” list) to help caregivers monitor required items, and a list of upcoming appointments (the Therapy Tracker) (Figure 2). Other My JAKE components included:

#### Medical/Developmental History

A comprehensive medical and developmental history filled out by caregivers during the screening/on boarding phase of the study and edited throughout.

#### The Autism Behavior Inventory (ABI)

An important element of My JAKE is the Autism Behavior Inventory (ABI), a web-based caregiver-rated scale for assessing ASD core diagnostic symptoms and associated behavior. The development of the ABI component of My JAKE have been previously published (Bangerter et al., 2017). The ABI consisted of 73 items divided into five domains: two ASD core symptom domains, SC, and restricted and repetitive behaviors (RRB); and three symptom co-occurrence domains, mental health (MH), self-regulation (SR), and challenging behavior. The ABI yields a score in each domain, plus a core score combining SC and RRB, based on behaviors that occurred within the last 7 days. The ABI can be downloaded in the USA from https://www.janssenmd.com/ (in the tools/psychiatry section) and accessed outside the USA via email request to autismbehaviorinventory@its.jnj.com. Questions were asked on two 4-point scales and consisted of either frequency and intensity ratings, frequency and context, or quality and context. The full ABI was completed by the primary caregiver at baseline, midpoint (Week 4), and endpoint. A short (35-item) form of the ABI (ABI-S) was completed by the investigator or delegate at baseline, Week 4, and endpoint. The primary caregiver completed the ABI-S on weeks when they were not required to complete the full ABI (Weeks 2, 3, 5, 6, and 7).

#### The Daily Tracker

The Daily Tracker consisted of several questions answered by caregivers of ASD participants. In this study, all caregivers were asked in the morning “How was (participant name)'s sleep last night?” and in the evening “How was (participant name)'s day?” Caregivers could also choose up to three additional behaviors to track, but only the two standard questions were analyzed. Questions could be answered by dragging a card along an 8-point scale ranging from “troubling” to “encouraging” either on the My JAKE mobile application or in a web browser. Responses to these questions were tracked and displayed over a 2-week period on the web site home page to provide feedback to caregivers and increase engagement in the study.

#### Mood Report

The affective circumplex model of affect reporting has a long and widespread history in measuring affective/emotional states, including those with ASD (Kring et al., 2003; Tseng et al., 2014). A digital version of the classical affective circumplex was created for the My JAKE mobile application for this study. The x-axis represents arousal, termed “activity,” and the y-axis represents valence, termed “mood.” The model was divided into “Quadrants” of activity and mood relationships. Caregivers moved the icon to a location on the screen that represented their child's perceived mood at the moment; they were asked to do this twice a day but could do so as many times as desired.

#### Event Tracker and Journal

Caregivers tracked all items of interest, including sleep and diet, on a daily basis. They were also able to provide free text entries *ad-hoc* to track both positive and negative events as they occurred.

#### Therapy Tracker

This module allowed tracking of participants' medical treatments or therapies, using a calendar-like interface. Caregivers had the option to export their created appointments to the calendaring system of their choice.

### Biosensor Data-JAKE Sense

JAKE Sense is based on the use of experimental biosensors to assess physiological characteristics and behavior related to core and associated symptoms of ASD. The biometric devices can be divided into two categories: lab-based biosensors, which measure biometric information in a laboratory setting (usually paired with challenge tasks or stimuli presented on a computer screen) and continuous biosensors, designed for daily wearable use to collect real-world biometric information about the participant. The selected biosensors (and their output) were used strictly for exploratory research purposes and were not used to diagnose, treat, or prevent any disease or disorder. All devices were used in compliance with their documented intended use.

### Biosensors

#### Continuous Biosensors

The “daytime biosensor” (Empatica E4™ Wristband) is a commercially-available wireless wristband biosensor that records EDA, skin surface temperature, blood volume pulse and inter-beat interval (producing an approximation of heart rate and heart rate variability), and 3-axis acceleration. The E4 has been used in previously published research (Goodwin et al., 2018) involving youth with ASD in the current study's participant age range (≥6 years of age) and did not require alterations to the band in either study to acquire quality signals. Both the EDA (Poh et al., 2010; Sano et al., 2014) and photoplethysmography sensors (McCarthy et al., 2016) in the E4 have been independently validated. It was worn during waking hours, at minimum for the afternoon until bedtime on weekdays, at periodic lab visits, and the entire day on weekends.

A “nighttime biosensor,” the commercially-available Ambulatory Monitoring, Inc. (AMI) Motionlogger® Actigraph was worn at bed-time for the entire night's sleep. Each night's sleep start time, number of awakenings during sleep, time duration of sleep, and sleep efficiency were derived from measurements recorded by the sensor (Acebo et al., 1999; Wiggs and Stores, 2004; Souders et al., 2009; Gringras et al., 2012; Meltzer et al., 2012), and have been validated in children with ASD.

#### Lab-Based Biosensors

These biosensors were used at a study visit while participants were exposed to specific visual or auditory stimuli or asked to perform a task (Table 3). Tasks and stimuli for use with lab-based biosensors comprised a battery of ~40 min for use at laboratory sites. Participants were asked to wear or use all the lab-based biosensors (in addition to the daytime continuous biosensor described above) during presentation of all tasks and stimuli. Tasks and stimuli were presented on a computer monitor using the iMotions® Biometric Research Platform (formerly called Attention Tool). The continuous and lab-based sensors were found to be tolerable (Ness et al., 2017).

**Table 3:** Tasks or stimuli (the JAKE Task Battery) for use with lab-based biosensors.

**Task/Stimulus**	**Description**	**Biosensor**				
		**Eye tracking**	**EEG**	**FACET**	**ECG**	**EDA**
Resting state-eyes open (Murias et al., 2007)	Video of sand falling through an hourglass for 1 min	x	*		x	x
Resting state-eyes closed (Murias et al., 2007)	Participant asked to close their eyes for 45 s		*		x	x
Event related potentials (Grice et al., 2005)	Static facial stimuli with averted or direct gaze	x	*		x	x
Social orienting task (Chawarska et al., 2012; Plesa-Skwerer et al., 2016)	Video of male or female actor presented in random order; actor engages participant in direct speech (dyadic bid) and joint attention, toward or away from a moving toy	*			x	x
Social vs. non-social videos (Pitcher et al., 2011)	Dynamic videos of children's faces (social) or toys (non-social)	x	*		x	x
Visual exploration task (Sasson et al., 2008, 2011)	Free viewing of Arrays of 24 images (including social images, HAI and LAI objects)	*			x	x
Biological motion (Umbricht et al., 2017)	2 side by side videos, in random left-right order. Each video contains dynamic point-light displays. One video is derived from human actor's performance; the other video is a computer-generated animation of moving dots	*	x		x	x
NimStim emotional faces (Wagner et al., 2013)	Static images of happy, angry, fear, and neutral faces	x	*		x	x
Activity monitoring (Shic et al., 2011, 2014; Umbricht et al., 2017)	Video recording of multiple human actors performing a social activity, with visually salient distracters in the background; actors focus on each other or on the activity only in 2 conditions	*			x	x
Funny videos	Funny videos, or videos designed to elicit an emotional response of surprise or joy			*	x	x
Expression of emotional faces	Participants asked to make faces to reflect basic emotions: Happy, Sad, Surprise, Scared, Angry, Yucky (disgust).			*	x	x
Auditory stimuli	3 sets of auditory stimuli (toilet flush, a ticking clock, or an 880 hz tone) presented for 3 s duration each, with ISI of 8–12 s each. Screen displayed bubbles screen saver and a progress bar indicating time until the presentation was complete.			x	*	*

* *primary hypotheses*.

x *additional hypotheses*.

*ECG, electrocardiogram; EDA, electrodermal activity; EEG, electroencephalogram; FACET, FACial Expression; HAI, high autism interest; ISI, interstimulus interval; LAI,low autism interest*.

Eye tracking data were collected using the Tobii X2-30 and synchronized with stimuli and facial affect data through the iMotions® eye tracking module.

EEG data were collected using the Brain Vision actiCHamp 32 and its proprietary software (BrainVision Recorder). A total of 20 electrodes were placed in accordance with the standard 10–20 electrode system. The use of a photodiode attached to the lower-right corner of the computer monitor allowed the software to precisely timestamp stimuli slide changes.

Electrocardiogram (ECG) data were collected using the CamNtech Actiwave Cardio Single-Channel ECG and used to assess heart rate and heart rate variability.

Facial affect detection was evaluated using the iMotions® FACial Expression Analysis (FACET) module and a high definition web camera.

**Figure 3:**
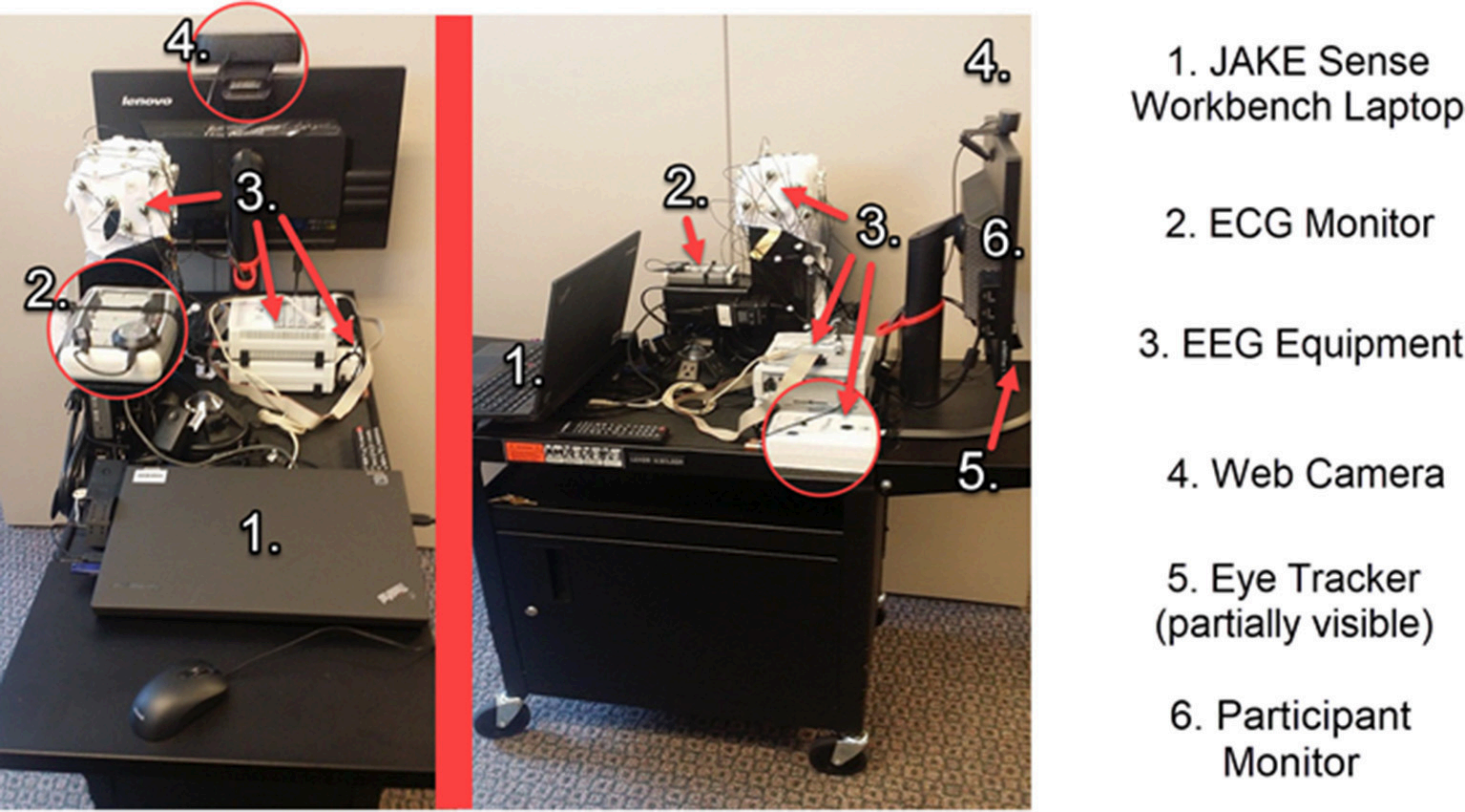
Assembled JAKE Sense workbench cart. ECG, electrocardiogram; EEG, electroencephalogram.

An assembled JAKE Sense setup is shown in Figure 3.

### JAKE Stream

JAKE Stream was designed to collect and process data from both My JAKE and JAKE Sense. JAKE Stream is divided into two physically distinct subcomponent sets of Microsoft.NET command line utilities hosted on Janssen servers: “My JAKE Data Pipeline” and “JAKE Sense Data Pipeline.”

The My JAKE Data Pipeline was designed to capture and process a daily archive of My JAKE data stored in a caregiver's and/or participant's Microsoft HealthVault account (described earlier). After the My JAKE Data Pipeline processed daily capture, data were directed to an internal Janssen server for traceability archiving/auditing and then ultimately to the Janssen data management team (via secure file transfer server) for incorporation into final study results. A site manager dashboard was used to view a read-only copy of My JAKE data stored on the internal Janssen server for monitoring participant, caregiver, and clinical site usage of web and mobile applications.

Similarly, the JAKE Sense Data Pipeline was designed to process and extract biometric features from data packages provided by JAKE Sense. Examples of the types of features that were extracted are shown in Table 4. After packages were archived to ensure traceability of derived analyses, a collection of MATLAB utilities scanned the packages to produce a series of extracted features (as flat.CSV-formatted files). Finally, the files were stored in a secured datastore server (“JAKE Sense Data Pipeline Features Archive”) for exploratory, proof-of-concept data mining; data are not used for clinical trial endpoints or for clinical decision-making.

**Table 4:** Example features.

**Eye-tracking**	**EEG**	**ECG**	**EDA**	**Actigraphy**	**FACET**
Tobii X2-30	Brain Vision ActiCHamp 32	CamNtech Actiwave Cardio Single-Channel ECG	Empatica E4™	AMI Motionlogger® Actigraph	iMotions® FACial Expression
General features • Fixations and saccades (using velocity based binocular algorithms) • % valid time on screen • % on ROIs (also normalized to % valid time) • Pupil size Specific features • Biological Motion preference (%), first saccade orienting (%) saccade latency, fixation orienting (%), fixation latency • VET exploration, preservation, detail orientation, RQA features	Induced EEG activity (estimated for each electrode and different brain region) • Power spectra at different bands (delta, theta, alpha, beta, gamma) • Normalized power spectra at different bands • Brain asymmetry index for different bands • Coherence between at different bands ERP • Components' amplitudes with peak- and area-based methods • Components' latencies with peak- and area-based methods	• HR • SDNN • SDSD • rMSSD • NN50 • pNN50 • ApEn • SpEn • LF HRV • Normalized LF HRV • HF HRV • Normalized HF HRV • LF/HF HRV	Tonic activity • SCL Phasic activity: • SCRR	• Sleep duration • Sleep start • Sleep end • # awakenings • Sleep efficiency	For each emotion (joy, anger, surprise, fear, contempt, disgust, sadness, confusion, frustration) and action unit defined according to FACS • Average evidence • Variance of evidence • Area under evidence curve

*ApEn, approximate entropy; ECG, electrocardiography; EEG, electroencephalograpy; EDA, electrodermal activity; ERP, event-related potentials; FACET, iMotions® FACial Expression analysis; FACS, Facial Action Coding System; HF, high frequency; HR, heart rate; HRV, heart rate variability; LF, low frequency; NN50, number of pairs of successive NNs that differ by more than 50 ms; pNN50, proportion of NN50 divided by total number of NNs); rMSSD, root mean square of successive differences; ROI, Region of interest (such as eye region, mouth region, face region, and so on); RQA, recurrence quantification analysis; SCL, skin conductance level; SCRR, skin conductance response rate; SDNN, standard deviation of normal to normal R-R intervals; SDSD, standard deviation of successive differences; SpEn, sample entropy; VET, visual exploration task*.

### Data Quality Control

All features extracted from data collected by JAKE were assessed and labeled according to their fidelity and quality (Webb et al., 2015), with analyses performed only on features reaching the following criteria:

As an automatic first step, EEG and eye tracking features were estimated on three levels, with data designated as “excellent,” “excellent+good,” and “any” quality. These three levels were defined as follows:

For eye tracking: “excellent” corresponds to the related value of calibration as defined by the iMotions® Biometric Research Platform software; “excellent+good” combines recordings with “excellent” and “good” calibration as defined by the software; and “any” corresponds to “excellent,” “good,” or “bad” calibration results as defined by the software.

For EEG: “excellent” corresponds to impedance below 25 kOhm; “excellent+good” corresponds to impedance below 50 kOhm; and “any” corresponds to any value of impedance. Additionally, we considered information from the eye tracker relating to times when participants were attending to the screen during EEG processing, allowing EEG segments corresponding to an absence of attention to be excluded from feature estimation. The use of “recalibration” videos embedded in the stimuli battery also allowed eye tracker calibration consistency to be checked throughout experiments. EEG data were further evaluated for eye-blinking artifacts and consequences of bridging electrodes. Corresponding EEG segments per channel (or data from a whole channel) were labeled accordingly.

Feature estimation algorithms incorporated information from these labels, wherein features were only estimated when no artifacts were identified. Artifact identification was performed by assessing mean, SD, maximum, minimum, median, mode, skewness, and kurtosis of EEG data values by applying sliding windows and comparing obtained distributions between channels with preset parameters in order to identify outliers. Since EEG follows 1/f distribution (pink noise) in the frequency domain, model slope, and model intercept for power spectrum density were estimated in sliding window segments and also checked for outliers via comparison of distributions between channels and with preset parameters. As a result of this artifact rejection step, diagnostic plots were created and visually inspected during subsequent manual quality checks.

After all features were automatically extracted, an expert manually checked the diagnostic plots produced during the automatic quality check including 1/f pink noise shape for spectra, presence of components for ERP, signal-to-noise ratio, detection of N peaks for ECG, and valid time for eye tracking to make additional quality determinations. This assessment resulted in modification of files with questionable data quality information and sometimes resulted in re-estimation of all features if necessary.

EDA data were manually and automatically inspected according to established guidelines (Dawson et al., 2007; Boucsein et al., 2012; Kleckner et al., 2018). This enabled identification of parts of recordings or whole recordings where data were missing (e.g., straight line at zero level), had poor signal quality, or were otherwise noisy. These values were subsequently excluded from analyses.

### Exit Interview

Caregivers were asked to provide final feedback on their experience with JAKE in an exit interview and a 36-question on-line survey. Examples of questions were “which JAKE components would you like to use outside of a clinical trial” and “how easy were the following tasks to complete.”

### Genomic Assessment

A genomic cheek swab sample was collected from ASD participants who consented separately to this component of the study. Participation in genomic research was optional.

### Safety Evaluations

All reported study events (intercurrent illnesses, changes in signs/symptoms, early discontinuations, device-related events, etc.) with onset during study participation were included in evaluations. For each event, percentages of participants who experienced at least one occurrence were summarized.

### Data Quality Assurance

Steps taken to ensure accuracy and reliability of clinical study data included selection of qualified investigators and appropriate study sites, review of protocol procedures with investigators and associated study-site personnel prior to study start, and periodic monitoring visits by the sponsor or their delegate.

### Data Analyses

All analyses performed for this study were exploratory. Pre-specified hypotheses relating to My JAKE and JAKE Sense were developed prior to conducting analyses. The overall Type I error rate for testing each hypothesis when two variables were assessed for correlation or the difference between ASD and TD participants involved a 2-sided or 1-sided significance level of 0.05. No adjustments for collinearity, multiplicity, or experiment-wise error were made as results were intended for hypothesis generation, not confirmation. Where presented, all confidence intervals are 2-sided at 95%, which correspond to a 2-sided significance level of 0.05 and a 1-sided significance level of 0.025. Descriptive statistics were provided for all study evaluations at baseline, Week 4, and study endpoint.

## Results

### Study Population

A total of 144 participants with a diagnosis of ASD were enrolled. Of these, 136 (94.4%) participants completed the study and 8 (5.6%) discontinued. The most common reason for discontinuation was self-withdrawal from the study (6 [4.2%]). The reasons for self-withdrawal were not reported. A total of 41 TD participants were enrolled and all (100%) completed the single visit of the study.

The ASD study population was primarily male (77.8%), consistent with higher male:female ratios for ASD (Loomes et al., 2017). The mean (SD) age of ASD participants was 14.6 (7.83) years compared with a mean (SD) age of 16.3 (13.18) years for TD participants. Mean (SD) ADOS total score of the ASD participants was 7.6 (1.7), their IQ was 99.2 (19.6), and all were verbal (Table 5).

**Table 5:** Participant characteristics.

**Characteristic**	**ASD *N* = 144**	**TD *N* = 41**
**GENDER**, ***n*** **(%)**		
Male	112 (77.8)	27 (65.9)
Female	32 (22.2)	14 (34.1)
**AGE**		
Mean (SD)	14.6 (7.83)	16.3 (13.18)
Median (Range)	12.5 (6–54)	11.0 (6–63)
**RACE**, ***n*** **(%)**		
White	118 (81.9)	34 (82.9)
Black or African American	6 (4.2)	2 (4.9)
Asian	4 (2.8)	0
Multiple	10 (6.9)	3 (7.3)
Other	4 (2.8)	0
Missing/unknown	2 (1.4)	2 (4.9)
ADOS CSS Total Score, mean (SD)	7.6 (1.7)	–
KBIT-2 IQ Composite Score, mean (SD)	99.2 (19.6)	–

*ADOS, autism diagnostic observation schedule; ASD, autism spectrum disorder; CSS, calibrated severity score; KBIT-2 IQ, Kaufman brief intelligence test, second edition; IQ, intelligence quotient; SD, standard deviation; TD, typical developing*.

### Data Capture and Quality

#### My JAKE

Overall, My JAKE was used an average of four times a day by each primary caregiver throughout the course of the study for over 34,000 distinct interactions.

As designed, My JAKE used at study sites captured the ABI with no missing items. On rare occasions, a few items were missing when a paper alternative was used due to internet failure at the sites.

The average (SD) number of reports for all participants for mood and overall type of day were 61.2 (35.9) and 29.8 (17.3), respectively, per caregiver over the course of the study.

#### JAKE Sense

Automated and manual curation of JAKE Sense data was used to assess amount and quality of data obtained from the various sensors and to exclude poor quality data from analyses. Table 6 shows the percentage of total features captured and the percentage of captured features classified as suitable for analysis (good) by sensor and experiment for ASD participants at the baseline visit using the current version of the data quality algorithms and feature extractors.

**Table 6:** Quantity and quality of data obtained from JAKE Sense biosensors (using current data quality algorithms and feature extractors).

**Sensor**	**Experiment**	**Features Captured (%)**	**Good Features (%)**
ECG	Eyes closed	97	98
ECG	Eyes open	94.8	97.9
EDA	Ticking clock	75.4	81.3
EDA	Toilet flush	75.4	83.8
EDA	Tone	76.3	80.1
EEG	BioMotion	80.5	60.7
EEG	ERP experiment	81.3	75.7
EEG	Social vs. non-social video	82.3	63.6
EEG	NimStim	80.3	45.6
EEG	Resting state	81.4	49.2
EYE	Activity monitoring	83.1	99.9
EYE	Biological motion	85.9	100
EYE	Social orienting task video	70.3	99.8
EYE	ERP experiment	86.7	100
EYE	Social vs. non-social video	91.1	100
EYE	NimStim	81.5	100
EYE	VET	89.9	99.9
FACET	Facial expression production	95.3	93.2
FACET	Funny videos	95.5	98.3

*ECG, electrocardiogram; EDA, electrodermal activity; EEG, electroencephalogram; ERP, event-related potential; FACET, FACial Expression; VET, visual exploratory task*.

The proportion of good features obtained (93.2–100%) was generally high for most of the experiments, although a lower overall proportion (45.6–83.8%) was seen in all EEG variables (biological motion, ERP, resting state, social vs. non-social videos, and NimStim) and EDA experiments (80.1–83.8%). Additionally, many of the EEG and EDA experiments had a relatively lower percentage of captured features than seen in the ECG, eye tracking, and facial affect experiments.

### Autism Behavior Inventory (ABI)

While full results of the ABI have been submitted as a separate manuscript, in short, the ABI performed well, with excellent test-retest reliability and strong correlations between domains and scales prospectively hypothesized to measure similar constructs (the core ABI Pearson correlation with the SRS-2 (0.81), ABI restrictive behaviors with RBS-R (0.77), ABI mental health with CASI-anx (0.78), ABI self-regulation with ABC hyperactivity (0.88), and ABI challenging behavior with ABC irritability (0.81). Conversely, as expected, lower correlations were observed where different symptom clusters were assessed, such as between the ABI challenging behaviors domain and the SRS-2 social communication and interaction score (0.32) (Table 7).

**Table 7:** Pearson correlations between ABI scales and subscales and related patient reported outcome measures.

**Analysis time point related PRO measure**	**Core ASD symptoms**	**Social communication**	**Restrictive repetitive behaviors**	**Mental health**	**Self-regulation**	**Challenging behavior**
**ABI SCALE**						
Baseline (*N* = 139)						
SRS-2 (Caregiver)						
Total score	0.81	0.65	0.74	0.50	0.47	0.35
Social communication and interaction	0.80	0.68	0.69	0.48	0.43	0.32
Restricted interests/repetitive behavior	0.71	0.46	0.76	0.49	0.52	0.41
CASI-Anx						
Anxiety scale score	0.54	0.34	0.58	0.78	0.38	0.25
RBS-R						
Overall score	0.68	0.40	0.77	0.45	0.52	0.42
ABC-Community						
Irritability/agitation	0.50	0.26	0.61	0.56	0.68	0.81
Lethargy/social withdrawal	0.70	0.69	0.52	0.35	0.17	0.19
Stereotypic behavior	0.60	0.40	0.64	0.36	0.53	0.42
Hyperactivity/non-compliance	0.44	0.23	0.54	0.30	0.88	0.54
Inappropriate speech	0.59	0.33	0.69	0.40	0.65	0.48

*ABC, Aberrant Behavior Checklist; ABI, autism behavior inventory; ASD, autism spectrum disorder; CASI-Anx, Child Adolescent Symptom Inventory – Anxiety; PRO, Patient Reported Outcome; RBS-R, Repetitive Behavior Scale – Revised; SRS-2, Social Responsiveness Scale 2; ZBI, Zarit Burden Interview*.

Test-retest reliability of each domain score 3 to 5 days after baseline was excellent, with Intraclass correlation coefficient values ranging from 0.85 to 0.95. Means did not change significantly between test and retest (Table 8).

**Table 8:** Test-retest correlations for all ABI subscales based on caregiver responses to ABI.

	**Core ASD *n* = 88**	**Social communication *n* = 87**	**Restrictive repetitive behaviors *n* = 88**	**Mental health *n* = 88**	**Self-regulation *n* = 88**	**Challenging behavior *n* = 88**
ICC estimate	0.91	0.91	0.88	0.85	0.92	0.95
*p*-value	< 0.0001	< 0.0001	< 0.0001	< 0.0001	< 0.0001	< 0.0001
95% CI	(0.88, 0.94)	(0.87, 0.94)	(0.83, 0.92)	(0.78, 0.90)	(0.88, 0.95)	(0.92, 0.97)

*p-value for difference from a one-sample t-test. Pearson correlation based on test and retest values. ICC was 2, 1 variant. ICC, intraclass correlation coefficient*.

### My JAKE

**Figure 4:**
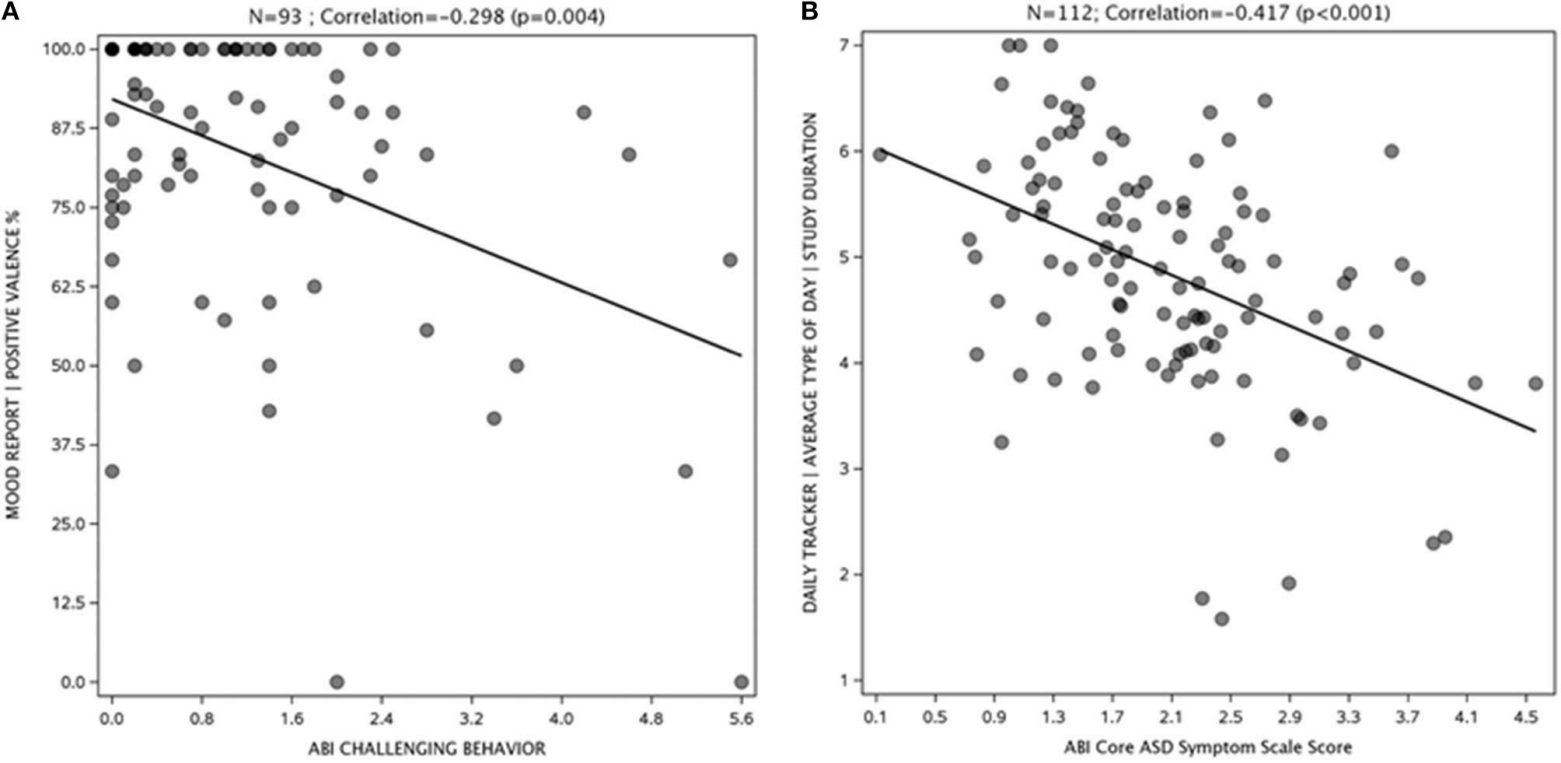
Mood report **(A)** and daily tracker **(B)** correlations with ASD symptoms. ABI, Autism Behavior Inventory; ASD, autism spectrum disorder; TD, typically developing.

Representative examples of results from My JAKE are shown in Figure 4. The mood report positive valence percentage showed a negative correlation with the ABI challenging behavior subscale at endpoint visit (*r* = −0.30, *p* = 0.004) indicating that more positively perceived mood correlated with a more favorable challenging behavior score (Figure 4A). Figure 4B illustrates that average perceived type of day negatively correlated with ABI Core score at baseline (*r* = −0.42, *p* < 0.001), indicating that a better day correlated with a better ABI Core score.

### JAKE Sense

**Figure 5:**
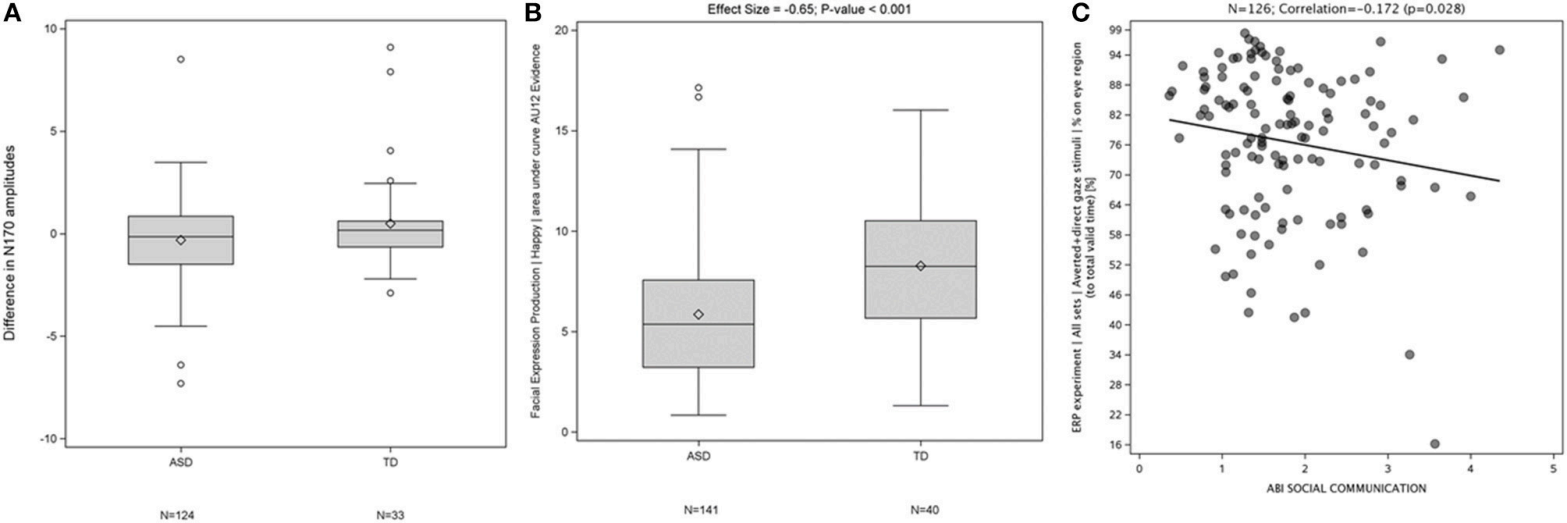
Difference in N170 amplitudes between ERP responses to direct and averted gaze stimuli at electrode T7 in TD and ASD participants (*p* = 0.053) **(A)**, Difference in production of “Happy” facial expressions between TD and ASD participants **(B)**, and Correlation between percentage of time spent by a participant looking at eye region (across both averted and direct gaze stimuli) corrected for total valid time (time on screen) and ABI social communication scales **(C)**. ABI, Autism Behavior Inventory; ASD, autism spectrum disorder; ERP, event-related potentials; TD, typically developing.

Examples of results generated by the EEG (A), FACET (B), and eye tracking components (C) of JAKE Sense are shown in Figure 5. The N170 amplitude (a measure of face processing in the ERP direct and averted gaze paradigm) displayed a difference between TD and ASD participants (Figure 5A). TD participants showed greater evidence of “happy” facial expression production in response to being asked to produce a happy face (Figure 5B). Also, the more time ASD participants spent looking at the eye region of images of faces in the direct and averted gaze paradigm, the better they scored on the ABI social communications domain (Figure 5C). Detailed presentations of results on FACET and eye-tracking are available elsewhere (Manfredonia et al., 2018; Manyakov et al., 2018) and manuscripts of additional results from these and other sensors are in preparation.

### Caregiver Feedback

**Figure 6:**
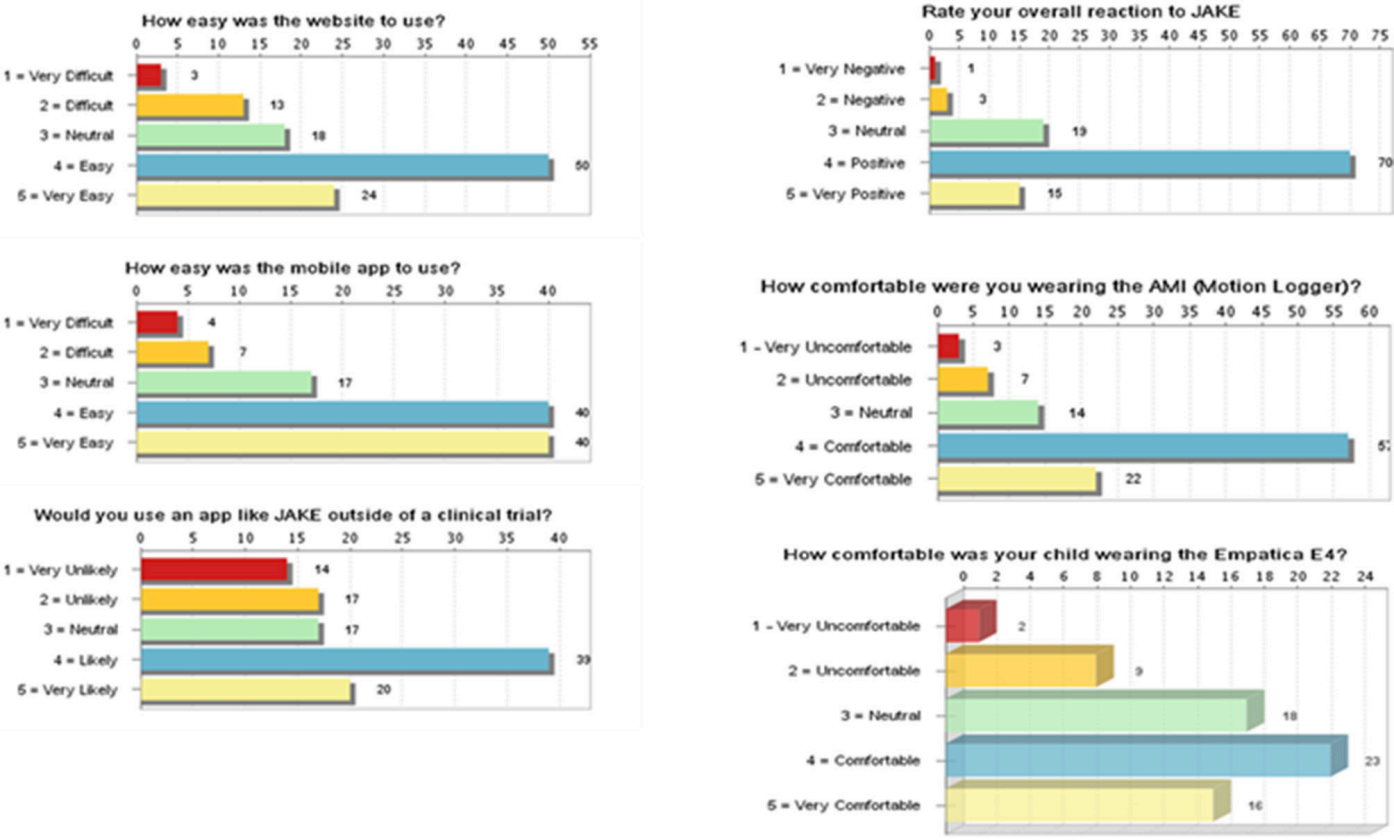
Results of exit survey completed by caregivers of children with ASD. ASD, autism spectrum disorder.

Most caregivers provided positive feedback on overall ease of use and utility using My JAKE for reporting and monitoring behaviors (Figure 6). Caregivers viewed the website as “easy” or “very easy” to use (69%, 74/108). Mobile application use by caregivers was rated as “easy” or “very easy” to use (74%, 80/108). Fifty-five percent (59/107) of caregivers were “likely” or “very likely” to use My JAKE outside of a clinical trial. Overall reaction to My JAKE was “positive” or “very positive” from 79% (85/108) of caregivers. For the majority of caregivers, their impression of use of the AMI Motionlogger actigraph by the participant was very comfortable (37%, 22/103) or comfortable (55%, 57/103). Participant's use of the Empatica E4 was reported as comfortable (34%, 23/68), neutral (26.5%, 18/68), or very comfortable (24%, 16/68).

### Safety

No significant device-related study events were reported. Approximately 40% of participants (57/144) with ASD had at least 1 event during the study (Table 9). The most frequently reported study events (≥1.0% of participants) were upper respiratory tract infection 6.9% (10/144), headache 4.2% (6/144), and nasopharyngitis 3.5% (5/144). No events were reported with the TD participants.

**Table 9:** Incidence of study events in ≥1.0% of participants.

	**ASD (*N* = 144) *n* (%)**	**TD (*N* = 41) *n* (%)**
Participants with at least one study event	57 (39.6)	0
Upper respiratory tract infection	10 (6.9)	0
Nasopharyngitis	5 (3.5)	0
Otitis media	3 (2.1)	0
Sinusitis	3 (2.1)	0
Gastroenteritis	2 (1.4)	0
Influenza	2 (1.4)	0
Respiratory tract infection viral	2 (1.4)	0
Urinary tract infection	2 (1.4)	0
Cough	2 (1.4)	0
Nasal congestion	2 (1.4)	0
Rhinorrhea	2 (1.4)	0
Headache	6 (4.2)	0
Migraine	2 (1.4)	0
Vomiting	3 (2.1)	0
Abdominal pain	2 (1.4)	0
Aggression	3 (2.1)	0
Seasonal allergy	4 (2.8)	0
Rash	2 (1.4)	0
Pyrexia	2 (1.4)	0

*ASD, autism spectrum disorder; TD, typically developing*.

Three of 144 ASD participants (2.1%) reported study events which were moderate or severe in intensity and designated as serious by the investigator. One participant reported an event of abdominal pain on study Day 40, which was of moderate intensity and resolved after 6 days. One participant reported an event of suicidal ideation on study Day 1, which was of severe intensity and resulted in psychiatric hospitalization. The participant was discontinued from the study due to this event. One participant reported two episodes of severe psychiatric decompensation. The first event occurred on study Day 23 and resolved after 8 days. The other event occurred on study Day 39 and resolved after 9 days.

## Discussion

The complexity and heterogeneity of ASD has contributed to difficulties finding effective and scalable therapies to treat core symptoms. Emerging tools and technologies have the potential to improve our ability to measure change more sensitively and to identify subgroups of individuals potentially responsive to novel therapies. In this study, we report on the practicality of combining and scaling multiple methodologies including biosensors and clinician and caregiver reporting to measure outcomes in a multisite study of children, adolescents, and adults with ASD. Recently, results from specific features of My JAKE and JAKE sense have been reported (Bangerter et al., 2017, In press; Manfredonia et al., 2018; Manyakov et al., 2018; Jagannatha et al., In press; Sargsyan et al., In press).

### Data Capture and Quality

An important feasibility metric is the amount and quality of data collected. Both JAKE Sense and My JAKE demonstrated high levels of performance as indicated by user feedback and the capture and quality metrics presented.

Capturing quality data in clinical trials for ASD is difficult due to cost and complexity, particularly when biosensors are included as outcome measures. Sample sizes are therefore often small, which prevents development of approaches for within-group stratification (Loth et al., 2017; Howes et al., 2018). There is a need for big data rich in features, gathered across a large sample, and at multiple levels across the same individuals (Lombardo et al., 2018). This study represents a successful attempt to gather this type of data in a large number of individuals with ASD across a range of levels of development and ages. Of particular importance is the number of sites involved (9) and the range of expertise of the sites in the use of biosensors (some sites had never used an EEG or eye tracker prior to this program), as well as the use of portable devices scalable to larger numbers of sites in a manner not prohibited by size or cost.

### JAKE Sense

The collection of hardware and software known as JAKE Sense enables collection of a wealth of synchronized data across multiple sensor modalities and stimulus paradigms. The ECG, eye tracker, and FACET sensors had minimal poor or missing data while data quality was more problematic for EDA and EEG.

Because of technical difficulties encountered in gathering EDA data from the home (for example connectivity issues) and the relatively lower quality of this data compared to other metrics when it was obtained at the sites, EDA has been removed from regular use in future studies within the JAKE system. Rather, participants will be given a single actigraphy device for continuous wear (day+sleep). EDA devices using adhesive wet electrodes may be another option for clinical studies.

The Brain Vision EEG collected reliable and valid data, but the proportion of test sets with acceptable data still needs improvement. Based on direct communication with study sites and analysis of session flow, a number of improvements are being implemented: (a) EEG will be integrated directly into the iMotions® Biometric Research Platform so as not to require separate recording software running simultaneously; (b) the process to ensure the photodiode is connected and functioning will be improved; (c) increased training in EEG setup and cap/electrode/impedance procedures, and (d) components of the EEG will be made more water-resistant to prevent damage.

Compared to our previous study where useable data was only gathered from ECG and FACET (Ness et al., 2017), the version of JAKE Sense used in this study, as described in Methods, represents a significant improvement in data collection. Even for EEG data which is often reported as more difficult to obtain (Gurau et al., 2017), and which had relatively lower success rates then our other sensors, substantial amounts of good quality data was obtained. Method changes that were implemented in this study such as better training of site personnel and change of EEG device contributed to the improved quality of data.

Based on feedback from sites and analysis of results, shortening the stimuli battery would also improve tolerability and attention of participants. Considering the length of various stimuli together with the proportion of high-quality data they provide, it is likely that ERP eye gaze, NimStim, and social vs. non-social video stimuli will be removed from the battery. Other tasks, for example biological motion, will be reduced in duration. “Funny videos” will be retained and refined, including the inclusion of sound, as these were reported to maintain interest of participants. The auditory task will be revised and an additional physical challenge (orthostasis) task will be added to specifically probe for changes in ECG measures. The result will be a task battery with two parts, each lasting < 15 min.

A novel ERP task that compares EEG response to social and non-social still images of HAI will also be added (Benning et al., 2016). ERP tasks are lengthy in nature, and the EEG set up is more arduous than other biosensors, but these assessments might indicate biological differences in ASD vs. TD individuals. Similar stimuli designed to elicit differences in brain-based responses are also a key component of other leading biomarker studies in ASD. For example, EU-AIMS includes EEG resting state and social and non-social videos in their battery of tasks (Loth et al., 2017). The ABC-CT, a longitudinal study of school-age children with ASD and typical development, is making similar efforts by evaluating a wide range of EEG and eye tracking biomarkers and their associations with caregiver-report and clinician-administered assessments of social communication skills (McPartland, 2018). A key distinction is the restriction of these studies to expert, academic sites. Furthermore, the ABC-CT focuses specifically on social communication skills as the primary endpoint, whereas JAKE includes a wider range of clinical endpoints, such as RRB, challenging behaviors, and mood.

### My JAKE

My JAKE was effective in engaging caregivers during the study and successfully captured data encompassing a broad range of demographic, medical, developmental, educational, and psychological aspects of ASD. The website and mobile applications were extensively used by caregivers throughout the study. Caregivers were prompted by My JAKE to complete the ABI and were able to use the web application to enter data on a full range of ASD symptoms.

### User Experience

Another important feasibility metric is user experience. Feedback from caregivers, both systematic (through the Exit Survey) and informal (through communication with study centers) was positive. The majority of caregivers found the system easy to use, helpful, and of interest to use again, even outside of a study. Though no major changes in My JAKE other than those discussed above are anticipated prior to deployment in interventional studies in autism, further refinements are planned, including improving charting features, enhancing the mood report, and modifying requirements for frequency of completing various components. These refinements are intended to increase acceptability to and use by caregivers.

To improve caregiver experience completing the ABI, analyses of data obtained in this study will focus on further decreasing user burden and simplifying scoring. Possible options under consideration include changing the number of anchors and altering the item filtering logic.

It is also anticipated that a decrease in duration of the JAKE Sense task battery will improve participant experience and tolerability. Consolidation of wearable home-based sensors to a single wrist-worn actigraph, rather than a different device for night time (actigraph) and day time (actigraph + EDA), may also improve compliance.

### Clinical Significance

Perhaps most importantly, clinical relevance of data acquired must be examined. We have reported on biosensor data analysis from facial expressions (Manfredonia et al., 2018) and eye tracking (Manyakov et al., 2018) and found both have the potential to aid with diagnosis and evaluation of clinical symptoms of ASD. Data mining methods of biosensor data were investigated as an approach to enable objective discrimination between ASD and TD individuals to potentially subgroup ASD participants based on phenotypic data gathered by JAKE (Jagannatha et al., In press). In addition, predictive modeling using biosensor-based feature selection was explored to examine how changes in these features correlated with clinical assessments (Sargsyan et al., In press). We are preparing additional results obtained by the various components of JAKE Sense, focusing on differences between TD and ASD participants' correlations between sensors and ASD severity and symptoms as measured by the ABI, and on changes over time. Genomic analysis will attempt to correlate polymorphisms in distinct genetic pathways with differences in autism phenotype.

In this study, we demonstrated the domains of the ABI were highly correlated with standard scales measuring the same constructs while comprising far fewer items than the combined battery of these scales. A complete presentation of results of the ABI from this study has been submitted for publication.

Similarly, the utility of My JAKE for capturing real-time daily reports was demonstrated, and further exploration of system elements and relationships of reports to other measures are underway. Clinical endpoints measured by components of My JAKE, such as the Daily Tracker and Mood Report, showed that even simple methods may be useful for measuring the effectiveness of interventional therapy.

### Limitations

Limitations of this study include a caregiver burden that was sometimes considerable, and the task battery being stressful for some participants. Technical failures with various sensors of JAKE Sense such as EDA and EEG also limited data capture. Improved algorithms for use in analyses of various sensor data and in data cleaning will also be explored.

A recent study (Anusha et al., 2018) suggests that dry electrodes on dry skin should be worn for at least 27 min before a sufficient moisture barrier is built up to produce stable EDA values. Although sites were requested to implement this warm-up time not all sites may have done so for all data collection sessions.

While a relatively heterogeneous group of individuals with ASD participated in the study, data from more individuals spanning autism severity and IQ is needed to determine whether there are meaningfully different profiles. This would necessarily require a much larger sample than allowed by this initial observational study. Also, individuals with ASD and particular symptoms, for instance prominent sensory sensitivities or inattention, may not have been able to tolerate portions of JAKE Sense.

Data were generally provided by the primary caregiver. It will be important to have data from other collateral sources such as school or program staff and more self-reported data from individuals with ASD who are capable of providing feedback on their own cognition, affect, and behavior.

As the primary purpose of the study was to assess the system in ASD participants, the size of the TD sample was relatively small in comparison. My JAKE was also not used by the TD cohort.

## Conclusions

JAKE is a dynamic system being developed to identify subpopulations of individuals with ASD and to sensitively measure ASD intervention outcomes. The use of JAKE in future interventional trials has the potential to advance novel treatments for ASD core and associated symptoms. In this study, JAKE demonstrated the ability to capture a broad range of high-quality data on ASD. Interventional studies will be needed to demonstrate the ability of JAKE to serve as a robust and sensitive measurement tool.

## Data Availability

The datasets generated for this study are available on request to the corresponding author.

## Author Contributions

SN, AB, JF, NM, DL, MB, AS, SJ, GD, MG, RH, BL, FS, YJ, BK, RT, JM, CS, and GP were involved in study design and/or data collection. DL, SJ, MC, and NM were responsible for the statistical analyses. SN, AB, NM, AS, and GP were involved in data analysis. All authors were involved in interpretation of the results and review of the manuscript.

### Conflict of Interest Statement

SN, AB, NM, MB, AS, SJ, MC and GP are employed by Janssen Research & Development, LLC and may hold company stock/stock options. DL was employed by Janssen Research & Development during the time of this study. GD is on the Scientific Advisory Boards of Janssen Research & Development and Akili, Inc., a consultant to Roche, has received grant funding from Janssen Research & Development and PerkinElmer, is CEO of DASIO (Digital Approaches Improve Outcomes), Inc., and receives royalties from Guildford Press and Oxford University Press. MG has received research and consulting funding from Janssen Research & Development. JF has received research grant funding from the NIH, Alcobra Pharma, F. Hoffmann-La Roche, Ltd., Fulcrum Therapeutics, Janssen Research & Development, and SyneuRX International. RH is on the Scientific Advisory Boards of BioMarin Pharmaceutical Inc., Neuren Pharmaceuticals Limited, and Janssen Research & Development, and has research grant funding from Curemark, Roche, Shire, and Sunovion Pharmaceuticals, Inc. BL has received research grant funding from the NIH, is a consultant to Janssen Research & Development, and the Illinois Children's Healthcare Foundation, and is a board member of the Brain Research Foundation. FS has received research funding from Janssen Research & Development, and Roche. BK, RT have received research funding from Janssen Research & Development, and Roche. The remaining authors declare that the research was conducted in the absence of any commercial or financial relationships that could be construed as a potential conflict of interest
